# Reverse transcription loop-mediated isothermal amplification (RT-LAMP) primer design based on Indonesia SARS-CoV-2 RNA sequence

**DOI:** 10.1186/s43141-023-00580-z

**Published:** 2023-12-18

**Authors:** Irsyad Ibadurrahman, Suryani, Desriani

**Affiliations:** 1 Biochemistry Department, IPB University, Jl. Raya Dramaga, Babakan, Bogor, West Java 16680 Indonesia; 2 Research Center for Genetic Engineering, Research Organization for Life Sciences and Environment, National Research and Innovation Agency, Jl Raya Cibinong KM 46, Bogor, West Java 16191 Indonesia

**Keywords:** COVID-19, Primer design, RT-LAMP, SARS-CoV-2

## Abstract

**Background:**

The COVID-19 pandemic has highlighted the importance of tracking cases by using various methods such as the Reverse transcription loop-mediated isothermal amplification (RT-LAMP) which is a fast, simple, inexpensive, and accurate mass tracker. However, there have been no reports about the development of RT-LAMP primer designs that use genome sequences of viruses from Indonesia. Therefore, this study aimed to design an RT-LAMP primer using SARS-CoV-2 genome sequences from Indonesia and several other countries representing five continents in the world, as well as genomes from five Variants of Concern (VOC).

**Result:**

The results showed that the consensus sequence of 70 SARS-CoV-2 virus sequences was obtained with a length of 29,982 bases. The phylogenetic test confirmed that the consensus sequence had a close kinship with the SARS-CoV-2 Wuhan Isolate. Furthermore, the SimPlot analysis showed that there was a high genetic diversity of sequences from the Coronaviridae tribal virus at base sequences of 1,500–5,000, 6,500–7,500, and 23,300–25,500. A total of 139 sets of primers were obtained from the primer design with 4 sets namely T1_6, T1_9, T4_7, and T4_52 having the best characteristics. Based on the secondary structure analysis test on 4 sets of primers, T1_6 and T1_9 were predicted not to form secondary structures at RT-LAMP operational temperatures. The primer set T1_9 showed better specificity in BLAST NCBI and eLAMP BLAST tests.

**Conclusion:**

This study obtained a primer set of T1_9 with base sequence F3: CACTGAGACTCATTGATGCTATG, B3: CCAACCGTCTCTAAGAAACTCT, F2: GTTCACATCTGATTTGGCTACT, F1c: GAAGTCAACTGAACAACACCACCT, B2: CCTTCCTTAAACTTCTCTTCAAGC, B1c: GTGGCTAACTAACATCTTTGGCACT, LB: TGAAAACAAACCCGCCGTCCTTG, which meets the ideal parameters and has the best specificity. Therefore, it is recommended for use in further tests to recognize SARS-CoV-2 from Indonesia, other five continents, as well as five VOCs, including the new Omicron sub-variant.

**Supplementary Information:**

The online version contains supplementary material available at 10.1186/s43141-023-00580-z.

## Background

Coronavirus disease 2019 (COVID-19) was declared by the World Health Organization (WHO) to cause respiratory distress. The disease was first discovered based on severe pneumonia symptoms observed in 44 droplet-borne patients in Wuhan, China. Other forms of nonspecific symptoms include pneumonia, acute respiratory distress syndrome (ARDS), organ dysfunction (sepsis), and septic shock [1]. These symptoms occur due to viral replication inside the human respiratory system. Infected lungs can cause a decrease in oxygen saturation in the body, leading to death. COVID-19 is caused by the SARS-CoV-2 (severe acute respiratory syndrome coronavirus 2) virus and was declared to be a pandemic on March 11, 2020.

The number of COVID-19 cases worldwide reported by the WHO has reached 230,418,451 positive cases with 4,724,876 deaths on September 24, 2021. According to WHO, the percentage of positive cases in each region of the world is divided into 38.5% in America, 30% in Europe, 18.6% in South and East Asia, 6.8% in the Eastern Mediterranean region, 3.5% in the Western Pacific region, and 2.6% in Africa. As stated by the Ministry of Health, Indonesian COVID-19-positive cases reached 4,204,116 on September 24, 2021, since it was first reported in March 2020. Furthermore, according to the Centers for Disease Control and Prevention (CDC), the number of positive cases can be obtained from diagnostic, screening, or public health surveillance testing (https://www.cdc.gov/coronavirus/2019-ncov/hcp/testing-overview.html).

COVID-19 testing in Indonesia is carried out through rapid diagnostic tests and RT-qPCR (reverse transcription-quantitative polymerase chain reaction). RT-qPCR uses viral nucleic acid as the basis for the examination and is the standard for COVID-19 testing [2]. The samples are obtained from the nasopharyngeal area and prepared in the laboratory for the extraction process of the virus’ genetic material. The extract of genetic material is then delivered into a special device called PCR that will duplicate the genetic material of SARS-CoV-2. Meanwhile, the rapid diagnostic test uses body proteins (antibodies) or viral proteins (antigens) as the basis for the examination [3]. Samples are in the form of blood plasma for rapid antibodies, or nasopharyngeal swabs for rapid antigens, and the result is only a line mark that indicates the presence of protein. These two types of COVID-19 testing still have various shortcomings.

RT-qPCR is considered a standard test to diagnose COVID-19 for good reasons. It has good specificity and sensitivity, a fast detection time within 2 h, advanced tools, and trained officers [4]. It is also the most valid test in diagnosing COVID-19 compared to other techniques [5]. In comparison, the rapid diagnostic test has the characteristics of a simple tool and a fast examination process, but poor specificity and sensitivity. It plays a role in tracking positive cases of COVID-19 but needs to be validated by RT-qPCR. On the other hand, RT-LAMP is a type of test based on the genetic material of the virus and has fairly good accuracy, a 30-min examination process, as well as an affordable price [6]. The Indonesian government through the Ministry of Health Decree Number HK.01.07/MENKES/4642/2021 has accepted RT-LAMP as a technique for COVID-19 testing (https://jdih.kemkes.go.id/). This technique is suitable for the rapid detection of COVID-19 cases and requires a good primer that fits the ideal criteria [7].

Primers are short strands of nucleotides that pair with the target DNA and play an important role in the doubling process. The polymerase enzyme will recognize the DNA primer and perform nucleotide amplification. Additionally, a DNA primer for RT-LAMP can consist of 2 pairs of internal, as well as one pair each of external and loop primers that are optional [8]. According to previous reports, the external primer base length is shorter (21–24 bp) than the internal (45–49 bp) [9]. RT-LAMP primers need to be designed in advance using various software on the computer such as Primer Explorer V5, genome-based LAMP primer designer (GLAPD), and FastPCR. Domestically and foreign-made RT-LAMP commercial products have been released to the Indonesian market due to the high need for COVID-19 detection. The results of the detection, according to the Indonesian head of the task force for handling COVID-19 (2022), are widely used for contact tracing, access to public facilities, as well as domestic and foreign travel. Although RT-LAMP products are already available, the potential for developing alternatives is still open. More importantly, there have also been no primer reports of domestic products because of commercial reasons importance.

In silico studies related to the primer design of RT-LAMP for COVID-19 testing have been carried out by Lu et al., Lamb et al., and Dong et al. [7, 10, 11], but they did not use genome sequences from Indonesia. Therefore, this in silico study was conducted to obtain primer designs using SARS-CoV-2 genome sequences from Indonesia and several other countries, as well as five variants of concern (VOC). WHO defines the VOC as having mutation variations from the Wuhan strain, and this viral infection significantly impacts global public health [12]. The results of this study can be used as an alternative to RT-LAMP primer for COVID-19 testing as well as a reference for future related studies.

## Method

In this study, in silico analysis was carried out using various bioinformatics applications.

### Sequence preparation and multiple sequence alignment

The 81 genome sequences of various SARS-CoV-2 variants from Indonesia and several other countries were downloaded from the GISAID database (https://gisaid.org) and listed in the supplement. Seventy sequences in the form of FASTA were analyzed with multiple sequence alignment MUSCLE in the Mega 11 application. MSA results are exported in the form of FASTA files. FASTA files are then consensus on the SnapGene Viewer application and stored in the FASTA file.

### Phylogenetic test

The consensus sequence of MSA results was used in phylogenetic analysis to determine kinship with viruses of the coronaviridae tribe using the Mega 11 application. The phylogenetic tree was created with the UPGMA method, 1000 bootstrap replications, a 2-parameter Kimura model, and a pairwise deletion option, while the results were saved in the PNG format.

### Determination of the primer design area

The consensus sequence of MSA results was opened on the SimPlot software, and the parameters used are the Kimura 2-Parameter model, windows size 1500 bp, and *step* 10 bp. SimPlot was run by selecting the SARS-CoV-2 consensus as a query in the “Commands” column and pressing “Start Scan.” The results were presented in the form of graphics saved in Bitmap format.

### Primer design

The RT-LAMP primer design was carried out through the Primer Explorer V5 web application (https://primerexplorer.jp/lampv5e/). The process consisted of 2 stages, the regular (F3, FIP, B3, and BIP) and the loop primer design (LF or LB). The ideal primer parameters set include the distance between the 5' F2 end primer and 5' B2 of 120–160 bp, the 5' F2 end with 5' F1c of 40–60 bp, and the 3' F3 end with 5' F2 of 0–60 bp. The ideal stability of the primer ends with a value of ΔG ≤  − 4 kcal/mol, a ΔG dimer value of >  − 2 kcal/mol, and a GC content ranging from 40 to 60%. The primer Tm value was estimated through the nearest neighbor method with experimental conditions set in the form of a concentration of Na ions of 50 mM, Mg ions of 4 mM, primers of 0.1 μM, and dNTP of 0.5 μM. In addition, Tm values for primers F1c and B1c ranged from 63 to 65 °C; F2, B2, F3, and B3 primers at 58–61 °C; and LF and LB primers at 63–65 °C.

FASTA sequences of target areas with a maximum base length of 2000 bp were entered in the “Target sequence file” section, and the manual parameter option was selected. Regular primer results were selected based on the values of the best primer parameters. The regular primer data were then downloaded in the form of a Primer Information File and were used to perform the loop primer design. This was carried out on the initial page of the regular primer design. The Primer Information file was included in the “Target sequence file” section, and the primer results of the loops were reordered based on the best parameter analysis. All primer data were saved through the “Save List” option.

### Primer sequence-structure analysis

Regular primers and best loops from the primer design were analyzed for the formation of hairpins, self-dimers, and heterodimers on the OligoAnalyzer Tool page (https://sg.idtdna.com/pages/tools/oligoanalyzer/). The parameters used in this analysis include ΔG values for the formation of hairpins, self-dimers, and heterodimers under certain conditions. The conditions of the analysis reaction were converted into 50 mM Na ions, 4 mM Mg ions, 0.1 μM primers, and 0.5 μM dNTP, while reaction temperatures were set at 25 °C constant. The formation of hairpins was also examined through further analysis on the RNAfold (http://rna.tbi.univie.ac.at/cgi-bin/RNAWebSuite/RNAfold.cgi). The designed primer sequence was inserted in the sequence column on the RNAfold page. The parameters used in this analysis were in the form of DNA options by Mathews [13], as well as temperatures of 25 °C and 60 °C, then the “proceed” button was clicked. The results obtained were predictions of the ΔG value of hairpin formation and its structure, while the primer set that passes the secondary structure test was further assessed using the specificity test.

### *Primer specificity test *in silico

The primer set of targets was analyzed using BLASTn on the NCBI database (https://blast.ncbi.nlm.nih.gov/Blast.cgi) and electronically simulated LAMP. The sequence of the primer set was written in query columns, and parameter changes were made. The adjusted parameters were in the form of adding a maximum target to 5000 and selecting the BLASTn algorithm in the “program selection” column, then SARS-CoV-2 species were added as search excludes. Species MERS-CoV, Bat-SARS-CoV, HCoV OC43, HCoV NL63, Influenza A, Human Respirovirus 1, Human Rubulavirus 2, Human Metapneumovirus, and Human Adenovirus were also added as search includes. BLAST analysis results were obtained in the form of species, percent identity, query cover, mismatch, and accession code. The last specificity test was carried out using the eLAMP program. The set of target primers and virus sequences that appear on BLAST was entered in the input field of the eLAMP. The parameters set include the minimum distance of the inner and middle primer of 15 bp, the minimum distance between the inner primer of 1 bp, the maximum distance of interloop space of 65 bp, and the maximum length of amplicons of 280 bp. The analysis results on the success of primer amplification were stored in CSV format, then the best DNA primer design was aligned to the new Omicron sub-variants, namely BA.1, BA.2, BA.4, BA.5, BA.2.12.1, BA.2.75, BQ.1, XBB, and XBC. All of the sequences were from Indonesia, except XBC which originated from Singapore.

## Result

### Sequence preparation and multiple sequence alignment

Sequence preparation is a process to ensure the sequence of organisms is downloaded in the form of a FASTA file. A total of 81 SARS-CoV-2 sequences were used including 30 from Indonesia as well as 51 from the African continent, America, Asia, Oceania, and Europe. Material supplements additionally show the variant types of the 81 SARS-CoV-2 sequences that have been downloaded. The sequences also include VOC and variants of interest (VOI) with various classifications. The base length of the downloaded SARS-CoV-2 sequences ranges from 28,918 to 31,649 bp.

The 70 SARS-CoV-2 downloaded sequences were analyzed in a multiple sequence alignment (MSA) process as the basis for the primer design. MSA is the process of computationally aligning sequences. There are a variety of MSA algorithms; in this study, we used the MUSCLE algorithm. MSA was used to obtain a consensus sequence with a base that could represent the 70 SARS-CoV-2 sequences, and the characteristics of the results are shown in Table 1. The base threshold for the nucleotide sequence had a percentage of 80%, indicating that each sequence of bases selected is the most dominant for SARS-CoV-2. Unidentified nucleotide bases also appeared in the consensus sequence and were represented by “N.” The fewer the number of unidentified bases, the better the consensus sequence.

**Table 1:** SARS CoV 2 consensus sequence analysis

Characteristic	
*Threshold*	80%
Base length	29,982 bp
Unidentified bases	315 bp

### Consensus sequence homology test

**Fig. 1:**
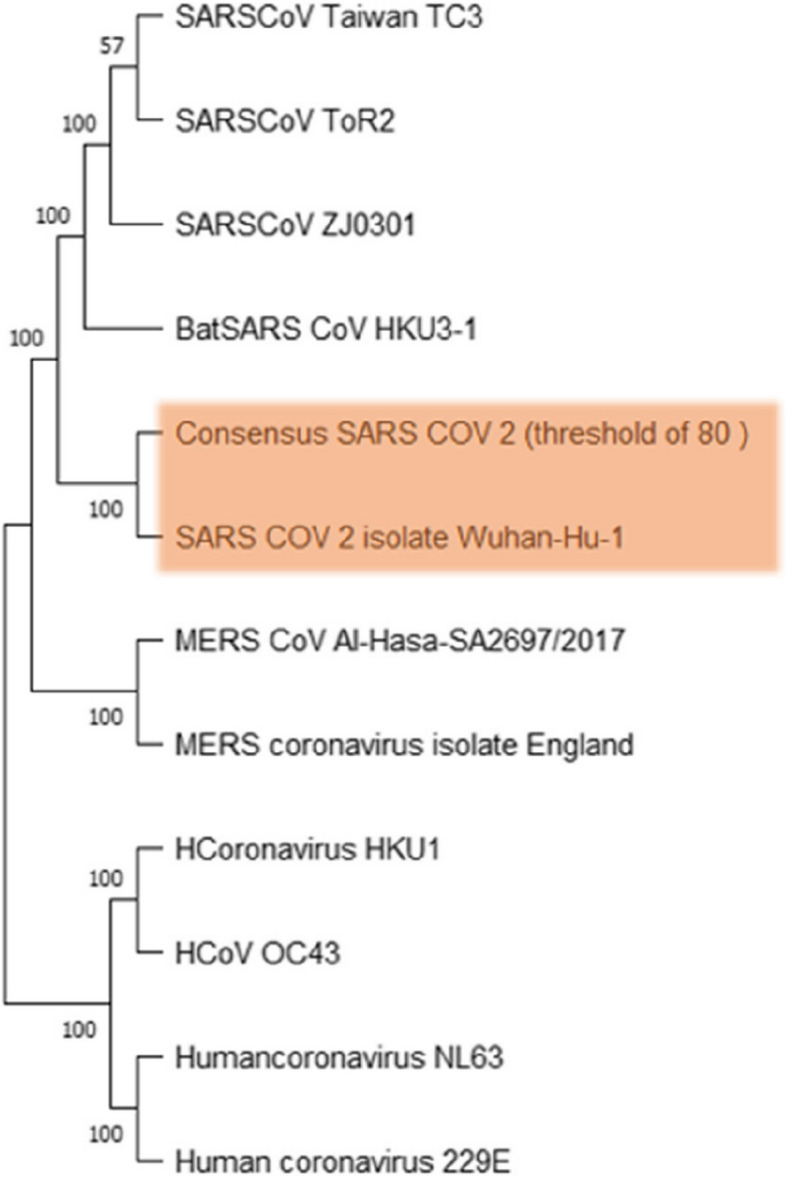
SARS CoV 2 consensus sequence cladogram

Homological analysis was performed to determine the kinship of the SARS-CoV-2 consensus sequence with other strains such as the Human Coronavirus (HKU1) sequence (OC43, NL63, and 229E), Bat-SARS-CoV, HKU 3–1, SARS-CoV (Taiwan TC3, ToR2, and ZJ0301), MERS-CoV (British isolate and Al-Hasa SA2697/2017), and SARS-CoV-2 Wuhan. Two clusters were obtained from the phylogenetic tree constructed as presented in Fig. 1. Accordingly, clade 1 consists of SARS-CoV, Bat-SARS-CoV, SARS-CoV-2, and MERS-CoV, while Clade 2 consists only of human coronavirus organisms. The cluster and branch formation result was also reinforced by the bootstrap value, wherein values close to 100 indicate the formation of a better tree model. The consensus sequence has the closest kinship to SARS-CoV-2 Wuhan. It is also closely related to Bat-SARS-CoV, SARS-CoV, and MERS-CoV but differs significantly from the human coronavirus.

**Fig. 2:**
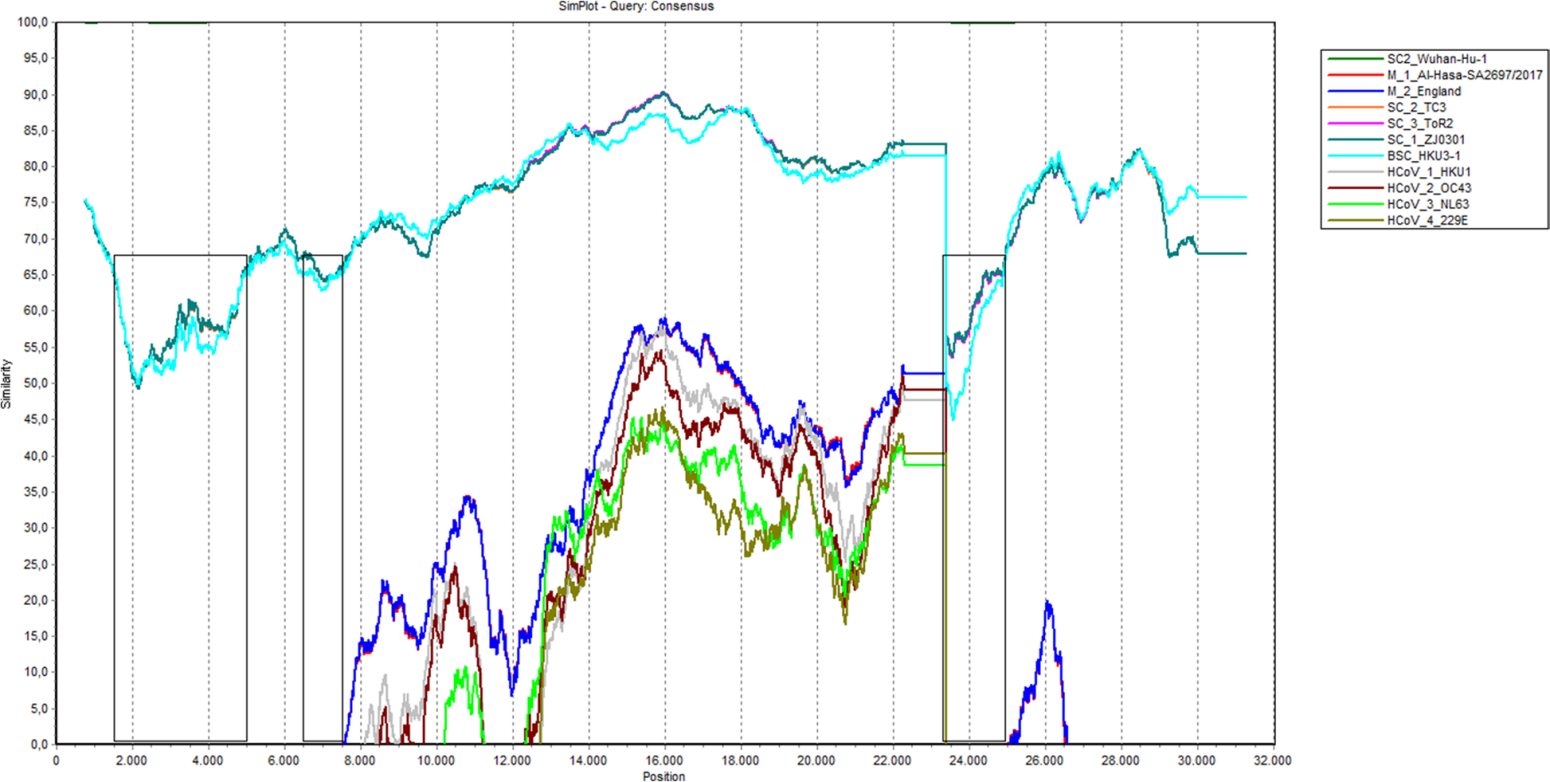
SimPlot analysis of the SARS CoV 2 consensus genome

The homology analysis was continued with SimPlot which aims to determine the target area of the primer design. The target area of the primer design is a sequence of sequence bases entered during the primer design process. SimPlot is an application that can display a similarity graph between each nucleotide base sequence of a query against the target sequence. Therefore, SimPlot can display the base range of the SARS-CoV-2 consensus sequence that has a low or high similarity to the sequence from the Coronaviridae Tribe. Figure 2 indicates that 3 design target areas were formed on the consensus sequence. There were 3 similarity patterns for the SARS-CoV-2 consensus sequence chart with organisms from the Coronaviridae Tribe. The highest similarity pattern was obtained with the SARS-CoV-2 Wuhan sequence which was around 99%. Meanwhile, the similarity pattern between SARS-CoV and Bat-SARS-CoV sequences ranged from 44 to 93%. A low similarity pattern was found for MERS-CoV and human coronavirus sequences namely < 64%. Based on the SimPlot analysis, the potential of the primer design target area is in the range of 1500–5000 bp, 6500–7500 bp, and 23,300–25,000 bp. This is because the area has a low similarity to the sequence in the medium and high similarity patterns. Therefore, 4 design targets with a maximum length of 2000 bp were obtained from 3 potential areas. The first target is a sequence with a position of 1500–3500 bp, while the second, third, and fourth were in a position of 3500–5000 bp, 6500–7500 bp, and 23,300–25,500 bp, respectively.

### Design primer

The primer design was performed through the Primer Explorer V5 page using 4 target area sequences, and the result showed that about 45 sets of primers were formed from target 1 and 94 sets from target 4, while no primer sets were formed for targets 2 and 3. The set of targets formed was then identified by observing the parameters of the length of the primer base, the percentage of GC, as well as the value of ΔG of the tip 5′, ΔG of the tip of 3′, and primer Tm. The results of primer identification based on these parameters showed the 4 best sets of primers. The order of bases is listed in Table 2, while Table 3 shows the characteristics of all the best sets of primers. Further selection of the optimal primer was based on other ideal parameter criteria. The secondary structures of the primers were identified using oligoanalyzers against the parameters of the hairpin, self-dimer, and heterodimer.

**Table 2:** The best primer base sequence of the SARS CoV 2 consensus sequence

Primer	Sequence 5′–3′
T1_6-F3	GCTTGTGAAATTGTCGGTGGA
T1_6-B3	AGTAGGCCAGTTTCTTCTCTG
T1_6-F2	CTGTGCAAAGGAAATTAAGGAGAG
T1_6-F1c	AGAGTCAGCACACAAAGCCAAAAA
T1_6-B2	ACACACTTTCTGTACAATCCCT
T1_6-B1c	TGGTGGAGCTAAACTTAAAGCCTTG
T1_6-LB	GTGAAACATTTGTCACGCACTCAA
T1_9-F3	CACTGAGACTCATTGATGCTATG
T1_9-B3	CCAACCGTCTCTAAGAAACTCT
T1_9-F2	GTTCACATCTGATTTGGCTACT
T1_9-F1c	GAAGTCAACTGAACAACACCACCT
T1_9-B2	CCTTCCTTAAACTTCTCTTCAAGC
T1_9-B1c	GTGGCTAACTAACATCTTTGGCACT
T1_9-LB	TGAAAAACTCAAACCCGTCCTTG
T4_7-F3	CCTGTTGCTATTCATGCAGATCA
T4_7-B3	CACCAAGTGACATAGTGTAGGC
T4_7-F2	TGGCGTGTTTATTCTACAGGTTC
T4_7-F1c	TGAGTTGTTGACATGTTCAGCCCC
T4_7-B2	TGGATTGACTAGCTACACTACGT
T4_7-B1c	CCATTGGTGCAGGTATATGCGCTAG
T4_7-LB	TCAGACTCAGACTAATTCTCTCGG
T4_52-F3	GTCAGAGTGTGTACTTGGACAAT
T4_52-B3	GTTACAAACCAGTGTGTGCCA
T4_52-F2	GTTGATTTTTGTGGAAAGGGCTATC
T4_52-F1c	AGAAGACTACACCATGAGGTGCTGA
T4_52-B2	TGAAACAAAGACACCTTCACGA
T4_52-B1c	GAACTTCACAACTGCTCCTGCCAT

**Table 3:** Best primer characteristic analysis results using Primer Explorer V5

Primer	Pjg. bases	% GC	Tm ^o^C	ΔG 5'	ΔG 3'
T1_6-F3	21	48	61.0	− 5.4	− 5.9
T1_6-B3	21	48	58.8	− 4.4	− 4.6
T1_6-F2	24	42	59.7	− 5.9	− 5.0
T1_6-F1c	24	42	63.7	− 4.6	− 4.0
T1_6-B2	22	41	59.2	− 5.2	− 5.1
T1_6-B1c	25	44	63.6	− 6.0	− 5.9
T1_6-LB	24	42	62.5	− 4.2	− 4.4
T1_9-F3	23	43	58.6	− 4.9	− 4.5
T1_9-B3	22	45	59.1	− 5.6	− 4.5
T1_9-F2	22	41	58.1	− 4.7	− 4.8
T1_9-F1c	24	46	63.1	− 4.4	− 5.8
T1_9-B2	24	42	59.4	− 5.3	− 5.3
T1_9-B1c	25	44	63.3	− 6.2	− 6.2
T1_9-LB	23	43	61.5	− 4.0	− 4.9
T4_7-F3	23	43	60.8	− 5.0	− 4.2
T4_7-B3	22	50	60.3	− 5.2	− 5.4
T4_7-F2	23	43	60.6	− 7.1	− 4.9
T4_7-F1c	24	50	65.4	− 4.4	− 7.0
T4_7-B2	23	43	60.5	− 4.4	− 4.9
T4_7-B1c	25	52	65.4	− 4.7	− 5.6
T4_7-LB	24	46	60.8	− 4.8	− 5.9
T4_52-F3	23	43	60.0	− 4.8	− 4.1
T4_52-B3	21	48	60.9	− 4.0	− 6.4
T4_52-F2	25	40	60.8	− 4.1	− 4.3
T4_52-F1c	25	48	65.2	− 4.1	− 5.7
T4_52-B2	22	41	60.0	− 4.2	− 5.6
T4_52-B1c	24	50	65.4	− 4.0	− 5.8

### Analysis of primer secondary structures

Table 4 presents the analysis result of the secondary structure on the Oligoanalyzer page which includes the ΔG value in testing the structure of the self-dimer, hairpin, and Tm value. The results showed that the ΔG value of self-dimer ranged from − 3.3 to − 9.9 kcal/mol, while the ΔG value in hairpin testing was between 0.4 and − 6.1 kcal/mol. The hairpin Tm is also below that of each of the primer Tm listed in Table 5 which shows heterodimer interaction testing ranging from ΔG values of − 3.6 to − 10.3 kcal/mol. Hairpin prediction by RNAfold using the minimum free energy method aims to compare the ΔG value of hairpin from an Oligoanalyzer at a temperature of 25 °C. The hairpin formed is the structure with the minimum free energy. The RNAfold analysis can only change temperature parameters, while the concentration of primer solution components including primer, Na ions, Mg ions, and dNTP has been established through Matthews’s (2004) parameter DNA. The prediction results in the form of ΔG hairpin values are listed in Table 6. The structures of the hairpin at temperatures of 25 °C and 60 °C are shown in Tables 7 and 8, respectively.

**Table 4:** Results of homodimer analysis using oligoanalyzer

Target	Primer	ΔG *Self* dimer	ΔG hairpin	Tm hairpin ^o^C
T1_6	F3	− 5.4	0.4	18.4
	B3	− 9.3	− 0.5	30.9
	FIP	− 8.4	− 4.6	57.5
	BIP	− 7.6	− 2.4	36.7
	LB	− 4.9	− 1.1	35.6
T1_9	F3	− 5.1	− 0.5	30.6
	B3	− 3.6	0.3	16.9
	FIP	− 8.8	− 3.5	50.4
	BIP	− 4.9	− 1.1	31.1
	LB	− 3.9	− 0.2	28.2
T4_7	F3	− 7.1	− 1.5	36.4
	B3	− 3.3	− 0.7	35.8
	FIP	− 8.1	− 3.3	42.7
	BIP	− 9.9	− 6.1	52.1
	LB	− 5.4	− 0.1	27.9
T4_52	F3	− 3.9	− 2.3	41.8
	B3	− 3.3	− 1.1	38.1
	FIP	− 6.4	− 4.0	64.7
	BIP	− 5.5	− 3.0	39.7

**Table 5:** The results of heterodimer analysis using oligoanalyzer

Target	Seq 1	Seq 2	ΔG	Seq 1	Seq 2	ΔG
T1_6	F3	B3	− 5.5	FIP	BIP	− 6.5
		FIP	− 7.2		LB	− 7.8
		BIP	− 6.7	BIP	LB	− 6.8
		LB	− 5.4			
	B3	FIP	− 88			
		BIP	− 7.8			
		LB	− 6.8			
T1_9	F3	B3	− 6.1	FIP	BIP	− 6.5
		FIP	− 5.5		LB	− 5.8
		BIP	− 5.5	BIP	LB	− 5.8
		LB	− 5.5			
	B3	FIP	− 7.0			
		BIP	− 7.0			
		LB	− 3.9			
T4_7	F3	B3	− 4.7	FIP	BIP	− 6.8
		FIP	− 8.0		LB	− 6.5
		BIP	− 7.0	BIP	LB	− 3.9
		LB	− 3.6			
	B3	FIP	− 6.2			
		BIP	− 10.3			
		LB	− 3.9			
T4_52	F3	B3	− 5.0	FIP	BIP	− 8.0
		FIP	− 5.6			
		BIP	− 5.4			
	B3	FIP	− 7.2			
		BIP	− 5.3

**Table 6:** The results of primer hairpin analysis using RNAfold

Target	Primer	ΔG hairpin at 25 °C	ΔG hairpin at 60 °C
T1_6	F3	− 0.16	0
	B3	− 1.30	0
	FIP	− 4.86	0
	BIP	− 4.05	0
	LB	− 3.00	0
T1_9	F3	− 0.79	0
	B3	− 0.07	0
	FIP	− 4.66	0
	BIP	− 1.90	0
	LB	− 0.48	0
T4_7	F3	− 2.09	0
	B3	− 1.22	0
	FIP	− 4.49	− 0.47*
	BIP	− 8.13	− 1.41*
	LB	− 0.36	0
T4_52	F3	− 3.67	0
	B3	− 1.08	0
	FIP	− 5.68	− 0.68*
	BIP	− 3.49	0

**Table 7:**
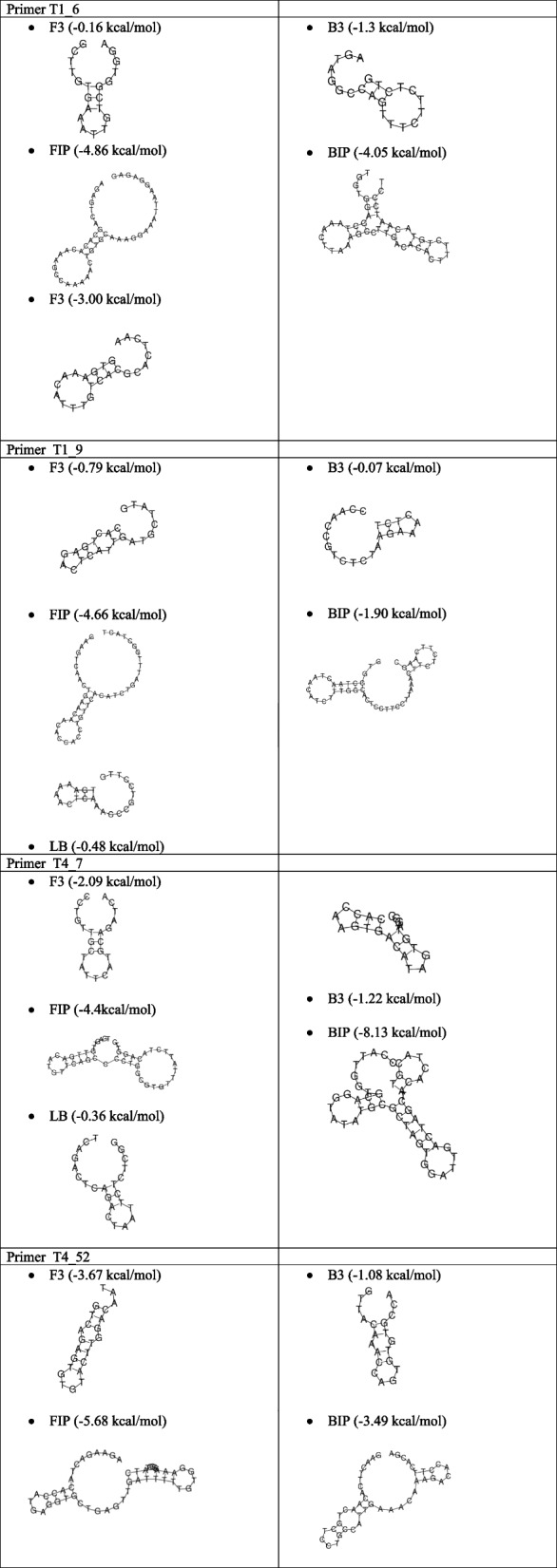
The structure of the primer hairpin at a temperature of 25℃

**Table 8:**
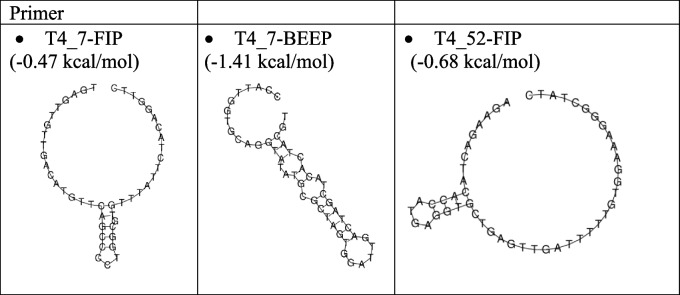
The structure of the primer hairpin at a temperature of 60℃

The RNAfold results showed that all target primers at a temperature of 25 °C had a value of Δ G hairpin ranging from − 0.07 to − 8.13 kcal/mol. A rise in the temperature to 60 °C caused a significant change in the structure of the hairpin. The RNAfold result of T1_6 and T1_9 primer sets did not form the hairpin structure while the T4_7 and T4_52 formed the hairpin structure. Moreover, the T4_7 and T4_52 primer sets had three hairpins with *G* values ranging from − 0.47 to − 1.41 kcal/mol. This shows that the hairpin structure was formed at the RT-LAMP reaction temperature for the T4_7 and T4_52 primer sets, while for the T1_6 and T1_9, no hairpin was formed.

### Primer specificity test

The T1_6 and T1_9 primer sets which did not form hairpins at a temperature of 60 °C were tested for specificity using the BLASTn NCBI and LAMP applications. Primer-BLAST test results in Table 9 showed that all primers identified as T1_6 have the potential to hybridize with 32 non-target sequences. Meanwhile, Table 10 shows that all identified primer T1_9 s can be attached to 31 non-target sequences. The values of the cover, percent identity, and mismatch queries generated on both primer sets also vary. The mismatch can be analyzed in total on a primer or at 5 bases from the 3′ end of the primer. The non-target sequence mismatch value at the end of 3′ is ≥ 3 bases, while the total mismatch value is expected to be ≥ 6 bases.

**Table 9:** The results of the primer set specificity test T1_6 with the BLAST method

Primer	*Query cover* (%)	*Identity* (%)	*Mismatch* (bp)		Potential hybridization	Accession
			End 3′	Total		
T1_6—F3 (21 bp)	100	100	0	0	Bat SARS CoV Longquan	KF294457.1
	100	95,2	0	1	Bat SARS CoV ZC45	MG772933.1
	85	100	1	3	HCoV NL63	DQ445911.1
	71	93.3	1	7^*^	MERS CoV	ON325308.1
	71	93,3	5^*^	7^*^	Influenza A	KX977705.1
	52	100	5^*^	10^*^	HCoV OC43	MK303624.1
	52	100	3^*^	10^*^	Human rubulavirus 2	MG836425.1
T1_6—B3 (21 bp)	100	95.2	0	1	Bat SARS CoV Longquan	KF294457.1
	100	95,2	0	1	Bat SARS CoV ZC45	MG772933.1
	76	93,8	0	6	Influenza A	KM027559.1
	66	100	1	7^*^	Human metapneumovirus	KJ627417.1
	61	100	3^*^	8^*^	HCoV NL63	MN306040.1
	57	100	2	9^*^	MERS CoV	MN481983.1
T1_6—F2 (24 bp)	100	100	0	0	Bat SARS CoV ZC45	MG772933.1
	70	94.1	2	8^*^	Influenza A	GU396969.1
T1_6—F1c (24 bp)	95	100	0	1	Bat SARS CoV Longquan	KF294457.1
	95	100	0	1	Bat SARS CoV ZC45	MG772933.1
	66	9 3.8	1	9^*^	Influenza A	MW872913.1
	50	100	0	12^*^	HCoV OC43	MN310476.1
	50	100	2	12^*^	Human metapneumovirus	AY525843.1
T1_6—B2 (22 bp)	100	100	0	0	Bat SARS CoV Longquan	KF294457.1
	100	100	0	0	Bat SARS CoV ZC45	MG772933.1
	72	93,8	5^*^	7^*^	Influenza A	MN319106.1
	54	100	2	10^*^	MERS CoV	MG987421.1
T1_6—B1c (25 bp)	100	100	0	0	Bat SARS CoV Longquan	KF294457.1
	100	100	0	0	Bat SARS CoV ZC45	MG772933.1
	52	100	5^*^	12^*^	Influenza A	MK424175.1
	52	100	3^*^	12^*^	Human adenovirus	KY002685.1
	48	100	5^*^	13^*^	HCoV OC43	OL770289.1
T1_6—LB (24 bp)	100	95,8	0	1	Bat SARS CoV Longquan	KF294457.1
	100	95,8	0	1	Bat SARS CoV ZC45	MG772933.1
	54	100	0	11^*^	Influenza A	MH592276.1

Description: ^*^ indicates a *mismatch* that meets the ideal criteria

**Table 10:** The results of the primer set specificity test T1_9 with the BLAST method

Primer	*Query cover* (%)	*Identity* (%)	*Mismatch* (bp)		Potential hybridization	Accession
			End 3′	Total		
T1_9—F3 (23 bp)	91	100	0	2	Bat SARS CoV Longquan	KF294457.1
	86	100	0	3	Bat SARS CoV ZC45	MG772933.1
	69	93,8	5^*^	8^*^	Influenza A	KU160859.1
	52	100	5^*^	11^*^	Human rubulavirus 2	MH892405.1
T1_9—B3 (22 bp)	100	100	0	0	Bat SARS CoV ZC45	MG772933.1
	95	90,5	1	3	Bat SARS CoV Longquan	KF294457.1
	72	93,8	0	7^*^	MERS CoV	ON325306.1
	59	100	5^*^	9^*^	Influenza A	L06576.1
	54	100	0	10^*^	Human metapneumovirus	MN745087.1
T1_9—F2 (22 bp)	100	95,5	1	1	Bat SARS CoV ZC45	MG772933.1
	100	95,5	1	1	Bat SARS CoV Longquan	KF294457.1
	72	93,8	5^*^	7^*^	Influenza A	MW848655.1
	54	100	3^*^	10^*^	Human adenovirus	KY002685.1
T1_9—F1c (24 bp)	66	93,8	1	9^*^	Influenza A	KF014012.1
	58	100	5	10^*^	MERS CoV	MN481986.1
	54	100	4	11^*^	Human adenovirus	OL450401.1
	50	100	5^*^	12^*^	HCoV OC43	MN310478.1
T1_9—B2 (24 bp)	100	87,5	0	3	Bat SARS CoV Longquan	KF294457.1
	83	90	1	6	Influenza A	OP023538.1
	50	100	4^*^	12^*^	MERS CoV	MG596803.1
	50	100	5^*^	12^*^	Human adenovirus	LC215437.1
	50	100	3^*^	12^*^	Human metapneumovirus	LC671557.1
T1_9—B1c (25 bp)	100	92	0	2	Bat SARS CoV ZC45	MG772934.1
	100	88	0	3	Bat SARS CoV Longquan	KF294457.1
	64	93,8	5^*^	10^*^	Human metapneumovirus	MH828686.1
	52	100	5^*^	12^*^	Influenza A	MT624534.1
	48	100	5^*^	13^*^	MERS CoV	MG987420.1
T1_9 – LB (23 bp)	69	93,8	5^*^	8^*^	MERS CoV	MF593268.1
	60	100	5^*^	9^*^	Influenza A	EU980459.1
	52	100	5^*^	11^*^	Bat SARS CoV ZC45	MG772934.1
	52	100	5^*^	11^*^	Human metapneumovirus	MH828687.1

Description: ^*^ indicates a *mismatch* that meets the ideal criteria

The BLAST method specificity test shows that the T1_6 primer set as a whole has the potential to attach to several non-target sequences with a low 3′ tip mismatch. Based on the results, the SARS-CoV ZC45 Bat Sequence has the highest potential to hybridize with all T1_6 primers. Longquan′s SARS-CoV-Bat sequence can be hybridized by 6 T1_6 primers including F3, B3, F1c, B2, B1c, and LB. These two sequences also have a total mismatch value and a low 3′ tip (< 3 bp). Sequences that can hybridize with T1_6 primers with low 3′ end and a total mismatch of 3 bp ≥ include Human Metapneumovirus, Human Rubulavirus 2, Human Adenovirus, MERS-CoV, Human Coronavirus NL63, and Human Coronavirus OC43 sequences. Based on the results, another non-target sequence that can hybridize with the primer is influenza A. Its sequences are known to be hybridized by all primers T1_6 with a total mismatch of 6 bp ≥ and a varying 3′ end mismatch.

The BLAST method specificity test also showed that the T1_9 primer set in general has the potential to attach to many non-target sequences and a high number of 3′ end mismatches with high non-target sequences. The SARS-CoV Longquan and Bat-SARS-CoV ZC45 sequences with the same accession code have a high potential to hybridize with 5 T1_9 primers. The 3′ end mismatch value on both sequences is categorized as low (< 3 bp). Furthermore, Influenza A. sequences in different isolates can be affixed by all T1_9 primers with a total mismatch of ≥ 6 bp. The 3′ end mismatch value is low in primer B2 and high in other primers. The T1_9 primer also hybridized with MERS-CoV, Human Metapneumovirus, Human Adenovirus, Human Coronavirus OC43, and Human Rubulavirus 2 sequences with a total mismatch of 7 bp ≥ and a high 3′ tip mismatch.

eLAMP is a program that aims to virtually simulate the Reaction LAMP, and primers suitable for the sequence tend to cause amplification. The analysis results are in the form of numbers that explain the amplification by the primer. As shown in Table 11, primers T1_6 and T1_9 can amplify all types of variants of SARS-CoV-2, consensus, and Wuhan sequence. However, amplification did not occur in SARS-CoV JZ0301, Bat-SARS-CoV ZC45, Bat-SARS-CoV Longquan, Influenza A, Human Metapneumovirus vzhmpvb2, Human Rubulavirus HPIV2i, MERS-CoV Riyadh-1758, Human Adenovirus case118_20131587, Human Coronavirus OC43, and Human Coronavirus NL63.

**Table 11:** The results of the primer set specificity test with the eLAMP method

Sequence name	T1_6				T1_9			
	F3	B3	FIP	BIP	F3	B3	FIP	BIP
SARS CoV 2 Consensus	1	1	1	1	1	1	1	1
SARS CoV 2 Wuhan	1	1	1	1	1	1	1	1
SARS CoV 2 Alfa EPI ISL 1904446	1	1	1	1	1	1	1	1
SARS CoV 2 Beta EPI ISL 2262295	1	1	1	1	1	1	1	1
SARS CoV 2 Delta EPI ISL 3761943	1	1	1	1	1	1	1	1
SARS CoV 2 Gamma EPI ISL 2493026	1	1	1	1	1	1	1	1
SARS CoV 2 Omicron EPI ISL 6795212	1	1	1	1	1	1	1	1
Bat SARS CoV Longquan	0	0	0	0	0	0	0	0
Bat SARS CoV ZC45	0	0	0	0	0	0	0	0
MERS CoV Riyadh-1758	0	0	0	0	0	0	0	0
*Human coronavirus* NL63	0	0	0	0	0	0	0	0
*Human coronavirus* OC43	0	0	0	0	0	0	0	0
*Human rubulavirus* HPIV2i	0	0	0	0	0	0	0	0
*Human metapneumovirus* vzhmpvb2	0	0	0	0	0	0	0	0
*Human adenovirus* case118_20131587	0	0	0	0	0	0	0	0
*Influenza A* Newyork-392	0	0	0	0	0	0	0	0

**Fig. 3:**
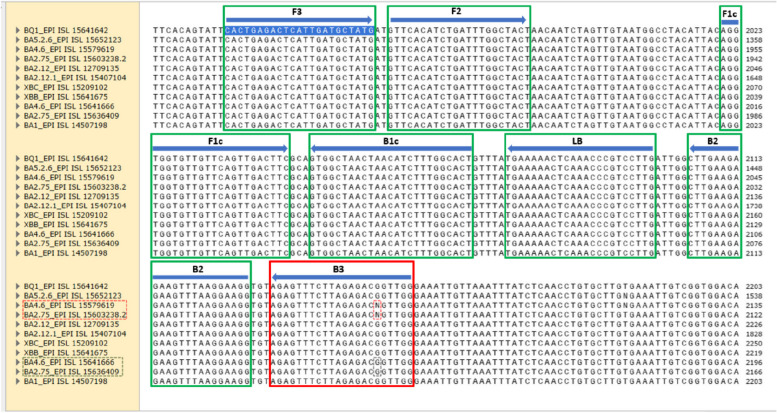
Alignment of T1_9 DNA primer in the new omicron sub-variant

In recent times, COVID-19 cases have increased again, specifically in Indonesia due to the new Omicron sub-variants which include BA.1, BA.2, BA.4, and BA.5. The best primer design in this study was then aligned to the new Omicron sub-variants. Based on the results, the primer set of T1_9 aligned with eleven sequences of Omicron sub-variants. In short, the B3 DNA primer showed one mismatch indicated by the letter N with accession code BA4.6_EPI IS 15579619 and BA2.75_EPI ISL 15603238.2 at base number six from the 3′ area (Fig. 3).

## Discussion

In general, a consensus sequence is generated by segregating and aligning multiple related sequences and then identifying the most common base at each position [14]. The sequence can come from DNA or RNA. Sequencing and alignment (MSA) are carried out using computational tools such as the Mega 11 application. There are two types of computational algorithms used by Mega 11, namely Multiple Sequence Comparison by Log-Expectation (MUSCLE) and Clustal Omega [15].

MSA algorithms have been extensively developed, with various examples such as MUSCLE, Fast Fourier Transform (MAFFT), T-Coffee, DIALIGN, KALIGN, Clustal Omega, and probabilistic consistency-based multiple alignments (PROBCONS) [16]. Among these, MUSCLE developed by Edgar [17] reportedly has higher accuracy and lower analysis time according to Mohamed et al. [16]. The quality of this algorithm is also not affected by the base length and the number of test sequences entered. This was stated after conducting a comparative analysis study with other types of MSA algorithms.

The consensus sequence obtained in this study has a base length characteristics of 29,982 bp, a threshold value of 80%, and 315 unidentified bases as shown in Table 3. The threshold is the limit value of the sequence in the selection of bases at each position. This suggests that it takes 56 of the 70 SARS-CoV-2 consensus sequences with the same base to produce one base at one position, hence, the higher the threshold value, the less variation in the consensus sequence. This value can also lead to an increase in unidentified bases when the sequences used have a high degree of variation. Unidentified bases tend to appear when there are gaps at the time of sequencing and alignment.

The creation of phylogenetics was carried out on the analysis of homology aimed at identifying the consensus kinship level of SARS-CoV-2 with the virus in the Coronaviridae Tribe. This was performed using the unweight pair-group with arithmetic means (UPGMA) method in the Mega 11 application. Aside from UPGMA, there is also a choice of Neighbour Joining methods, Maximum Likelihood, Minimum Evolution, and Maximum Parsimony in the application. The UPGMA method calculates the distance matrix of two organisms and the smallest p-distance index between pairs is used to combine organisms in 1 clade [18]. Additionally, this method is the oldest and the simplest, and it also considers the evolution along the tree of phylogeny to be constant [19].

Based on the phylogenetic tree-making results, the consensus sequence of SARS-CoV-2 is in one clade with SARS-CoV-2 from Wuhan, and this is appropriate because both sequences are of the same species. The cladogram results also showed that the SARS-CoV-2 clade has the same ancestor as the SARS-CoV sequence and the SARS-CoV HKU 3–1 Bat, hence, all three species are taxonomically included in the sarbecovirus subfamily. The consensus sequence on the cladogram indicates differences between the clade and the MERS-CoV sequence, but they both have the same origin. This occurred because MERS-CoV is taxonomically included in the *Betacoronavirus* genus but is classified again in the sub-genus. Meanwhile, the human coronavirus sequence is in a different clique because it belongs to the Alphacoronavirus clan, which is separate from other sequences. All the results of this kinship analysis correspond to the taxonomy of the virus presented in this study [20].

In addition to the formation of the clade, the value of the bootstrap is also a reinforcer of this kinship analysis. It is generated to test the confidence of the clade model in phylogenetic analysis and the results showed that only the SARS-CoV Taiwan TC3 and SARS-CoV ToR2 nodes had a bootstrap value of 57. This value is categorized as good, and the clade formed can be considered acceptable. The threshold for rejection of clade formation is a bootstrap value of 50, which implies the potential for bias [21]. Therefore, the cladogram results of this phylogenetic assay are acceptable and there is no indication of bias.

SimPlot is an application developed by Stuart Ray in 1998 and used to plot the similarity of bases to their position in a sequence. The basis of its analysis is to calculate and plot the percent of identities from query to reference sequences, then the results are visualized in the form of lines on a new window. The analysis is not only a stand-alone program but can also be found in conjunction with other applications. T-RECs are an example of applications that includes SimPlot as one of the bases for its analysis. The application can perform large-scale and fast recombinant detection [22]. Originally, SimPlot was first used as a method by Lole et al. [23] to identify the recombination of human immunodeficiency virus type-1. Then, Utama and Shimizu [24] also used this application to determine the similarity between poliovirus and the coxsackie A virus. Currently, SimPlot is being used as a genome similarity analysis tool for the Wuhan-Hu-1 SARS-CoV-2 sequence against various sequences from Coronavirus [25]. This shows that SimPlot analysis has been used in various studies including in the genome analysis of SARS-CoV-2.

SimPlot analysis in this study aims to identify alkaline regions in the SARS-CoV-2 consensus sequence that have a low level of similarity with those from the Coronaviridae tribe. The results showed 3 similarity patterns on the SimPlot chart. The first pattern is SARS-CoV-2 Wuhan sequence which had a high similarity value of > 99%. A similarity pattern was also obtained for the SARS-CoV and Bat-SARS-CoV sequence chart patterns which range from 44 to 93%. Meanwhile, the MERS-CoV and human coronavirus sequences showed a low similarity pattern of < 64%. The three similarity patterns showed potential target areas and were in the nucleotide base position of 1500–5000 bp, 6500–7500 bp, and 23,300–25,000 bp. This is because the area has a low level of similarity to the SARS-CoV-2 consensus sequence.

SARS-CoV-2 encodes 11 polyproteins in its life cycle [20], and the genes can be identified in the potential target area. The base position of 1500–7500 bp in the consensus sequence is the region of the gene encoding non-structural protein 3 (nps3), while protein spikes are encoded at 23,300–25,000 bp.

To design a set of RT-LAMP primers, six to eight primers are needed to produce amplicons from the target sequence; hence, it is necessary to identify the best characteristics and primer selection. The characteristics of the best primer candidates are divided into two, namely ideal for each primer and also for the set [9, 24]. Each primer needs to have a base length, melting temperature (Tm) value, GC content, 3′ stability value, and a corresponding secondary structure. Meanwhile, each set of primers needs to have a distance between the primer regions, a Tm balance, and a corresponding secondary structure. The design and identification of the primer have been carried out and 4 sets of candidates with the best characteristics were obtained.

The base length of each primer suggested by Patel and Sarma [26] is ideally 18–24 bases, while Soroka et al. [9] suggested 21–24 bp for external primer (F3/B3) and 45–49 bp for internal primer (BIP/FIP). The longer the primer, the longer it takes for the primer to hybridize or detach from the molded sequence [26]. Based on the results, the 4 best sets of primer candidates had a range of base lengths from 21 to 25 bp. Candidates with a base length of 25 bp are primer B1c from T1_6, B1c from T1_9, B1c from T4_7, as well as F2 and F1c from T4_52.

According to Zhang and Tanner [27] who tested the quality of RT-LAMP primers for the detection of SARS-CoV-2 in vitro and obtained 5 out of 18 primer sets with a base length of > 24 bp. The sensitivity test results showed that 2 sets of primers were included in the good category, 2 sets in the moderate, and 1 in the bad. This shows that primers with a base length of 25 can still be used and are of fairly good quality. Therefore, primer candidates T1_6-B1c, T1_9-B1c, T4_7-F2, T4_52-F2, and T4_52-F1c can be considered acceptable for use. The GC percentage is the ratio of the base content of guanine (G) and cytosine (C) found in the primer sequence. This value affects the primer binding on the molded sequence [28]. The base pair of GC has more hydrogen bonds namely 3, than AT bonds; hence, a high %GC can strengthen the primer bond with the mold, but extremely high values will complicate the release of the primer and increase the probability of secondary structure formation [29]. The recommended %GC value for primer ranges from 40 to 60%. Based on the identification results, the primer candidates had a %GC ranging from 40 to 52%, which is an ideal %GC.

The stability of the primer end is the strength in binding the mold and is influenced by the base composition of the GC also known as the GC clamp determined at 5 bases from the primer end. Good stability ΔG values at both ends of the primer should ideally be between − 4 and − 8 kcal/mol [30]. The results showed that the primer set candidates corresponded to the ideal value of primer end stability as indicated by their ΔG values which ranged from − 4 to − 7.1 kcal/mol. Furthermore, the number of GC clamps or ΔG of 3′ end stability needs to be considered. This is because the 3′ end is the initial site of alkaline elongation of the polymerase enzyme; hence, the more stable it is, the easier the sequence amplification.

The Tm value is the temperature used to denature 50% of DNA double strands and primer pasting [31]. Values above or below the ideal criteria may reduce the effectiveness of the hybridization or cause the primer to be easily mis-primed, respectively [32]. Based on the results, the Tm value produced by the primer candidate had a temperature that range from 58.1 to 65.4 °C, while the ideal Tm value is between 55 and 65 °C. This shows that some primers have Tm values higher than the ideal range with a different value of < 0.4 °C. Non-suitable primer candidates are T4_7-F1c primers, T4_7-B1c, T4_52-F1c, and T4_52-B1c. These Tm values can be changed and optimized during in vitro primer tests, one of which is by changing the concentration of the reaction solution on RT-LAMP.

The primer secondary structure is formed due to interactions between the nitrogenous bases. This structure is divided based on the location of the interaction, namely intermolecular and intramolecular. The intramolecular secondary structure is also called the hairpin structure, while the intermolecular consists of a self-dimer and a heterodimer [33]. Additionally, hairpins are characterized by the shape of loops and stems formed on their structure. Then, self-dimer is a secondary structure formed from a bond between the same two primers, while a heterodimer is formed from two different primers. All these secondary structures need to be avoided as they can cause the poor attachment of the primer to the mold or block the binding site to the enzyme.

One parameter that needs to be observed to avoid secondary structures is the Gibbs energy value (ΔG). The more negative the ΔG, the stronger the bond, and the greater the energy needed to break the structure [34]. Therefore, secondary structures with extremely negative values of ΔG should be avoided. The ΔG value measurement and the prediction of secondary structures also need to pay attention to the factors of temperature, composition, and concentration of the solution. Oligoanalyzers and RNAfolds are applications that predict the structure and value of hairpin ΔG. The temperature factor is known to be irreversible on Oligoanalyzer analysis, while the RNAfold can be changed. Therefore, the RNAfold is used to supplement the analysis of the Oligoanalyzer.

The hairpin structure analysis data with Oligoanalyzer showed that all primer candidates were predicted at a temperature of 25 °C, ΔG values ranging from 0.4 to − 6.1 kcal/mol, and Tm between 16.9 and 57.5 °C. According to a previous study, the tolerance limit of the hairpin ΔG value is >  − 3 kcal/mol [31]. The RNAfold analysis results confirmed that at a temperature of 25 °C, all primer candidates were predicted to form hairpins with ΔG values ranging from − 0.07 to − 8.13 kcal/mol. At a temperature of 60 °C, only 3 primers formed the hairpin, namely the T4_7-FIP, T4_7-BIP, and T4_52-FIP primers. Consequently, this is the most common temperature used for the RT-LAMP reaction [35–37]. Based on the results, T4_7 and T4_52 primer sets are not recommended for use as SARS-CoV-2 RT-LAMP primers.

Other secondary structures that need to be identified are self-dimers and heterodimers. The analysis with Oligoanalyzer showed that all primer candidates were predicted to form self-dimer and heterodimer structures. Ideally, the ΔG value of dimerized primer should be >  − 9 kcal/mol according to Ghosh et al. [33]. The analysis results showed that 3 primers had a ΔG value <  − 9 kcal/mol. The T1_6-B3 and T4_7-BIP primers have self-dimer ΔG of − 9.3 and − 9.9 kcal/mol, respectively, while the T4_7-BIP primers have heterodimer ΔG values with B3 of − 10.3 kcal/mol. These ΔG values were calculated at 25 °C and might change to be more positive at higher temperatures.

The Tm balance value is the difference between candidate primers in a set, and the results showed that the candidates for each primer set had a difference in Tm values of 4.9 oC for T1_6, 5.2 °C for T1_9, 5.1 °C for T4_7, and 5.2 °C for T4_52. According to Borah (2011) [38], the ideal difference in the Tm value should be < 5 °C, to avoid amplification failure. Another study conducted by Zhang and Tanner [27] tested the quality of RT-LAMP primers for in vitro detection of SARS-CoV-2. One of the primers used, namely N2, had a difference in Tm values of 6 °C, but it can be amplified and produce good sensitivity. Therefore, the 4 primers set candidates are acceptable for use even with a difference in Tm values of 5.2 °C.

The distance characteristics between the primer regions of the four primer set candidates are already specific and have been computationally identified in the Primer Explorer V5 application. This is because the distance between the regions needs to be adjusted when inputting primer design parameters. This includes the distance between the 5′ F2 end primers with 5′ B2 of 120–160 bp, the 5′ F2 end with 5′ F1c of 40–60 bp, and the 3′ F3 end with 5′ F2 of 0–60 bp. The distance between these primer regions follows the ideal value [39].

BLAST is a program developed by the NCBI database to identify local similarities in sequences. The program also uses the same techniques as the Mega 11 application, namely sequencing and alignment (MSA). BLASTn is one of the features in the BLAST program intended for nucleotide search. The analysis results are in the form of the species name, max score, total score, query cover, *E* value, accession length, accession code, summary graph, and alignment [40]. These characteristics can be used for various purposes, especially for homological and specificity analyses [41]. This is because primer specificity analysis can be obtained through the parameters of the cover, percent identity, and mismatch queries.

Furthermore, query cover is a ratio that describes the alignment of query and target sequences. A query cover of 100% is achieved when the target sequence base includes the entire query sequences. Percent identity is the ratio of the number of bases in the target sequence identical to the query sequence. Mismatches can be manually calculated by adding the number of unsorted primer bases and those that are not identical to the reference sequence; hence, the higher the mismatch value, the better the primer in terms of specificity. This study used BLAST analysis to identify primers that can cross-react with non-target sequences namely respiratory viruses and Coronaviridae tribes. The primer sets T1_6 and T1_9 were used in the specificity analysis because they did not form the structure of the hairpin and passed other ideal parameters.

The specificity analysis results showed that the primer set of T1_6 and T1_9 could cross-react with non-target sequences. All primers T1_6 were found to cross-react with varying mismatch values. They consistently cross-react with the SARS-CoV Longquan and Bat-SARS-CoV ZC45 bat sequences with a total value and a 3′ mismatch tip of ≤ 1 base. This low mismatch value on non-target sequences needs to be avoided because it reduces primer specificity. Some T1_6 primers can also attach to sequences from MERS-CoV, Influenza A, Human Metapneumovirus, Human Adenovirus, HCoV OC43, and HCoV NL63 with a total mismatch value of ≥ 6 bases. This high total mismatch value against non-target sequences is necessary because it increases the specificity of the primer. A total mismatch of ≥ 6 bases can reduce the amplification efficiency, thereby preventing the formation of amplicons with mold sequences [42].

The primer set T1_9 can cross-react with non-target sequences, for example, F1c and LB primers can attach to non-target sequences with high total values and 3′ mismatch ends. The match of 2–3 bases at the 5 end of the 3′ PCR primer requires Taq polymerization enzymes for the amplification process [43]. This suggests that a mismatch > 3 bases at the 3′ end can lead to the failure of the mold amplification process by the primer. Based on the results, T1_9-F1c and LB primers have good specificity compared to T1_9-F3, B3, F2, B2, and B1c. Longquan and ZC45 SARS-CoV Bat sequences were also easily affixed by 5 of the 7 T1_9 primers and had a mismatch value of ≤ 3 bases. Some T1_9 primers can attach to sequences from MERS-CoV, Influenza A, Human Metapneumovirus, Human Adenovirus, HCoV OC43, and HCoV NL63 with high mismatch values, indicating the high specificity of the primer set T1_9.

The T1_9 primer set is considered to have better specificity compared to the T1_6 set. This is because fewer T1_9 primers were attached to the SARS-CoV Longquan and Bat-SARS-CoV ZC45 sequences. These two sequences can attach several times to both sets of RT-LAMP primers produced. The T1_9 is also known to have a high mismatch value with non-target sequences, while the BAT sequence of SARS-CoV has a high similarity with SARS-CoV-2. Furthermore, the phylogenetic analysis and SimPlot show that the Bat-SARS-CoV and SARS-CoV have high proximity to the SARS-CoV-2 consensus sequence. According to a previous study, the genetic similarity between SARS-CoV-2 and Bat-SARS-CoV ZC45 is 87.8% [26]. This indicates that the lower the potential for primer hybridization, or a high mismatch value with Bat-SARS-CoV, the better the primer specificity.

eLAMP is a software program that can perform a virtual simulation of LAMP reactions by matching the primer set of conjectures on the printed sequence [44]. The program uses base matching techniques, primer 3’ mismatches, and distance measurements between regions to perform specificity analyses [43]. In this study, the primer set was tested with both SARS-CoV-2 and sequences from BLAST analysis results that had the potential to stick to the primer. The specificity test using the eLAMP method showed that the primer set was amplified by the SARS-CoV-2 sequence. This is because both sets of primers are formed from the base sequence of SARS-CoV-2 at base positions 1500–3500. The primer set did not amplify non-target sequences presumably due to mismatches or non-conformities in the distance of attachment. In addition, the eLAMP analysis showed that the primer set of T1_6 and T1_9 was specific to SARS-CoV-2 and no amplification with non-target sequences occurred.

In light of the increasing COVID-19 cases worldwide due to the new Omicron sub-variant, the best primer design, T1_9 set primer, was also aligned with this new sub-variant to confirm its suitability for detection. Based on the results, only the B3 DNA primer showed one mismatch with the new Omicron sub-variant (with accession code BA4.6_EPI ISL 15579619 and BA2.75_EPI ISL 15603238.2) specifically at base number 6 from the 3′ area. Since the mismatch was indicated with the letter N, and was also not present in other sub-variants, it was possible that there was no mismatch in this area. This implies that the best primer design will perfectly recognize the new Omicron sub-variant. Moreover, since the mismatch occurred only once and far from the 3′ area, it is unlikely to significantly affect the DNA primer’s ability to recognize the target.

## Conclusion

This study was conducted to design and test primer sets for the RT-LAMP method to detect SARS-CoV-2 in silico using 81 sequences from Indonesia and other 5 continents. In conclusion, the primer set T1_9 with a base sequence F3 CACTGAGACTCATTGATGCTATG, B3: CCAACCGTCTCTAAGAAACTCT, F2: GTTCACATCTGATTTGGCTACT, F1c: GAAGTCAACTGAACAACACCACCT, B2: CCTTCCTTAAACTTCTCTTCAAGC, B1c: GTGGCTAACTAACATCTTTGGCACT, and LB: TGAAAAACTCAAACCCGTCCTTG was found to meet the ideal criteria in terms of %GC, primer length, melting temperature (Tm), ΔG primer end stability, lack of a secondary structure, and high specificity.

### Supplementary Information

**Additional file 1:** **Table 1.** Accession codes, variant types, and region origins of 81 SARS CoV 2 sequences.

## Abbreviations

RT-LAMP: Reverse transcription loop-mediated isothermal amplification
SARS-CoV-2: Severe acute respiratory syndrome coronavirus 2
COVID-19: Coronaviruses disease 2019
RT-PCR: Reverse transcription polymerase chain reaction
MSA: Multiple sequence alignment
VOC: Variant of concern

## Declarations

### Availability of data and materials

All data generated or analyzed during this study are included in this published article (and its supplementary information files).
