# Supplementary material for: Reverse transcription loop-mediated isothermal amplification (RT-LAMP) primer design based on Indonesia SARS-CoV-2 RNA sequence
Source: J Genet Eng Biotechnol. 2023 Dec 18;21:168. doi: 10.1186/s43141-023-00580-z (PMC10728416; doi:10.1186/s43141-023-00580-z)
Supplement: Supplementary file 1 — Additional file 1: Table 1. Accession codes, variant types, and region origins of 81 SARS CoV 2 sequences. [file 43141_2023_580_MOESM1_ESM.docx]

Table 1 Accession codes, variant types, and region origins of 81 SARS CoV 2 sequences

| Types of variants | | Region | | | | | |
| --- | --- | --- | --- | --- | --- | --- | --- |
|  |  | Indonesian | Afrikaans | America | Asia | Oceania | Europe |
| VOC | Alfa | EPI_ISL_1416191, EPI_ISL_5328539 | EPI_ISL_3717929,  EPI_ISL_1760623 | EPI_ISL_3761572, EPI_ISL_3501666, EPI_ISL_3669472 | EPI_ISL_2500849, EPI_ISL_2460317 | EPI_ISL_1904446, EPI_ISL_3011179 | EPI_ISL_3734369, EPI_ISL_2636562 |
|  | Beta | EPI_ISL_1824605, EPI_ISL_2262295, EPI_ISL_2382409, EPI_ISL_3138796, EPI_ISL_538500 | EPI_ISL_3472802, EPI_ISL_3730476 | - | EPI_ISL_3614345, EPI_ISL_2536954, EPI_ISL_2779639 | EPI_ISL_1424505, EPI_ISL_2920953, EPI_ISL_3568461 | EPI_ISL_3274382 |
|  | Gamma | - | EPI_ISL_2493026 | EPI_ISL_2778008, EPI_ISL_3708858, | EPI_ISL_1589853, EPI_ISL_1969692, | EPI_ISL_1416322, EPI_ISL_1406435 | EPI_ISL_3631610, EPI_ISL_3713461 |
|  | Delta | EPI_ISL_3138888, EPI_ISL_3691664, EPI_ISL_3755966, EPI_ISL_2854743, EPI_ISL_6262544, EPI_ISL_3550537, EPI_ISL_3691602, EPI_ISL_2931787, EPI_ISL_6827377, EPI_ISL_1824604, EPI_ISL_3691643, EPI_ISL_3691668, EPI_ISL_3691680 | EPI_ISL_3150949,  EPI_ISL_3547696 | EPI_ISL_3537362, EPI_ISL_3760728, EPI_ISL_3769528, | EPI_ISL_3315874, EPI_ISL_3717776, EPI_ISL_3305850 | EPI_ISL_3722805, EPI_ISL_3707626 | EPI_ISL_3761943, EPI_ISL_3768862 |
|  | Omicron | EPI_ISL_14507198  EOI_ISL_12709135  EPI_ISL_15579619  EPI_ISL_15641666  EPI_SIL_15652123  EPI_ISL_15407104  EPI_ISL_15603238.2  EPI_ISL_15636409  EPI_ISL_15641642  EPI_ISL_15641675 | EPI_ISL_6704875 | - | EPI_ISL_15209102 | - | EPI_ISL_6795212 |
| VOI | Lamda | - | EPI_ISL_3355716 | EPI_ISL_1366175 | - | EPI_ISL_1494722 | EPI_ISL_5136632 |
|  | Mu | - | EPI_ISL_4920267 | EPI_ISL_2135033 | - | - | EPI_ISL_1683896 |
